# From Social Network to Peer Support Network: Opportunities to Explore Mechanisms of Online Peer Support for Mental Health

**DOI:** 10.2196/41855

**Published:** 2023-02-28

**Authors:** Amy Rayland, Jacob Andrews

**Affiliations:** 1 Academic Unit of Mental Health and Clinical Neuroscience School of Medicine University of Nottingham Nottingham United Kingdom; 2 National Institute for Health and Care Research Mindtech Medtech Co-operative Institute of Mental Health University of Nottingham Nottingham United Kingdom; 3 National Institute for Health and Care Research Nottingham Biomedical Research Centre Institute of Mental Health University of Nottingham Nottingham United Kingdom

**Keywords:** peer-to-peer support, Facebook, social networking sites, mental health, moderation

## Abstract

An increasing number of psychological interventions are shifting to online modes of delivery. One such intervention is peer-to-peer support, which in this context may provide internet users living with mental health disorders an opportunity to connect with and support others living with similar conditions. This paper presents a call for further research into how platforms such as Facebook could be used as channels for peer support and the mechanisms that may underlie their effectiveness. We discuss the background of peer support, how it has transitioned online, and consider theories and models that may have relevance. We also consider the importance of moderation within online peer support and the development of specific social network–based online interventions. We conclude that for social network sites to be used as peer-to-peer support interventions, more research is needed to understand their effectiveness, the role of moderation in these communities, and the mechanisms that produce the benefits experienced by users.

## Introduction

Mental illness can be described as “health conditions that involve changes in emotions, thinking or behavior (or a combination of these)” [1]. Mental illnesses range from “common mental disorders” (CMDs), such as depression and anxiety, to psychotic and personality disorders [2]. Research indicates that 1 in 6 adults in England had a CMD in 2014 [3], and recent evidence suggests that the effects of the COVID-19 pandemic have exacerbated mental illness, with almost 1 in 5 adults in the United Kingdom experiencing some form of depression in 2021 [4]. Furthermore, 16% of children younger than 16 years were identified as having a probable mental health disorder in the same period [5].

Mental illnesses have a significant impact on quality of life, social functioning, work, and family activities. Many of those living with mental illness have educational difficulties, being less likely to finish school or enter college [6]. This is compounded by social and relationship problems, vulnerability to abuse, and significant social stigma and discrimination. Aside from the individuals themselves, family members of those with a mental illness often take on caring responsibilities and experience chronic stress from the emotional burden of care. It is estimated that mental illnesses cost the UK economy at least £117.9 billion (US $141 billion) annually (amounting to around 5% of the United Kingdom’s gross domestic product), with almost three-quarters of this being attributable to the loss of productivity and the costs associated with unpaid informal care [7].

As the demands for mental health services exceed physical resources, it is more important now than ever that individuals are able to access free and widely available support. The use of social media is widespread across the world, with use in developing countries exceeding that in developed countries [8]. The growth of social media has seen it being used for many different reasons, including communication, marketing, advertising, media sharing, and entertainment. More recently, as the widespread accessibility of the internet for clinical interventions has been noticed, there has been an increased interest in using social media to deliver online interventions for mental health. Facebook, as the most popular social media platform [9], provides all the necessary tools to make it a suitable location for peer-to-peer support groups. However, as this is an emerging area of research, little is known about the effectiveness of Facebook-mediated peer support or what potential mechanisms may underlie any behavioral and attitudinal changes observed as a result of its use.

This viewpoint paper considers the background to peer support, how it is currently being used online, and the scope for Facebook to be used in this area, as well as suggesting some models and theories that may help to explain the mechanisms underlying effective online peer support. We suggest next steps in research to enable the development and rollout of online peer-to-peer support interventions for the benefit of those living with mental illness.

## Traditional Peer Support

Peer support within mental health can be defined as support or services provided to individuals experiencing mental health problems by others who have experienced similar problems [10]. Peer support is not based on psychiatric models or diagnostic criteria but can be understood as an extension of the natural human tendency to respond compassionately to shared difficulty [11]. Peers, seen as equals, use their lived experiences to provide “been there” empathy, insight, encouragement, and assistance and to inculcate hope in a reciprocal relationship [11,12].

The roots of peer support services lie in long-established groups, such as Alcoholics Anonymous [13], designed to build upon peer support that occurs naturally between people [14]. Mental health service providers recognized the benefits of these groups and have since introduced peer support programs across the United Kingdom [13], with peer workers employed in various roles, including facilitating mutual support groups, providing one-to-one support, or running alternative services [15]. As services are provided by nonprofessionals, they can be available in a community setting at a relatively low cost compared to professional services.

The effectiveness of peer support has been examined in relation to multiple outcome measures. A recent meta-analysis of 28 randomized controlled trials investigating the effectiveness of peer support for individuals with a range of mental illnesses demonstrated significant improvements in clinical recovery (ie, measures of psychiatric symptoms) and personal recovery (ie, measures of perceived recovery, sense of purpose, and personal agency) but not functional recovery (ie, measures of quality of life and the degree of vocational and social functioning) [16]. Functional recovery did, however, show significant improvements for those with serious mental disorders. Peer support also has a positive impact on psychosocial and recovery outcomes [15], which include increased community integration, sense of control, social functioning, and social support; peer support also promotes feelings of empowerment and hope [17-20]. Furthermore, peer support can improve self-esteem, self-efficacy, and self-management of difficulties [21].

Despite evidence supporting their effectiveness, these formal services are usually only available through mental health services, which are facing pressures due to cuts in funding [22] and demand exceeding available National Health Service resources [23].

## Online Peer Support

Recent figures suggest that 99% of people aged 16 to 44 years in the United Kingdom are internet users, with 89% of UK adults reporting using the internet daily [24]. There is a growing body of evidence demonstrating the benefits of internet interventions for mental health conditions, including depression and anxiety [25]. Interventions delivered online eliminate geographical barriers, can be accessed and used at any time, and may be more cost-effective than traditional services for young people aged 12 to 25 years [26].

Peer support can be provided informally online. This support is more informal in that it is not tied to services and can be accessed by anyone on the internet. Asynchronous platforms, such as forums, discussion groups, and bulletin boards, allow users to post topics and engage in discussions, using these platforms to exchange knowledge, ask for help, discuss problems, and share stories [27]. Synchronous platforms, which can be text-based chat rooms or purposely created virtual worlds, host real-time communication between users [28].

A systematic review indicated that internet support groups (ISGs) are most often used for emotional and informational support (more often experiential knowledge than “second-hand” professional knowledge), which creates a sense of social companionship [29]. This review found that 10 of 16 single-intervention or cross-sectional trials showed a positive effect of ISGs on depression symptoms, although study quality was rated as low, and the studies covered a range of mental and physical health conditions [30]. In one of these studies, which specifically examined individuals with depression, those who were more frequent users of the ISG (defined as using the group for 5 or more hours per week) showed a higher likelihood of depression resolution than less frequent users [30].

A review of online peer support for individuals with depression suggested several benefits, including increased empowerment and self-efficacy, enhanced coping strategies, and reduced social isolation [31]. However, there was limited empirical evidence to validate these descriptive findings. In a systematic review of online peer support for adolescents and young adults, Ali and colleagues [32] found mixed results, with only 2 of the 6 studies included in the review showing evidence of a positive effect. These 2 studies demonstrated reductions in anxiety [33] and higher abstinence from smoking [28] after using online peer support. A further review of digital peer support for mental health found early evidence of feasibility, acceptability, and effectiveness among all studies, though it reported that the studies were lacking in measurements of fidelity, limiting understanding of underlying mechanisms [34].

Qualitatively, many advantages of online synchronous peer support platforms have been identified, including emotional support, the availability of advice, enablement of positive personal changes, engagement in valuable social interactions, and the opportunity to disclose and express feelings and views [35,36]. Furthermore, it has been suggested that online forums can function as platforms for peer education—being “experiential experts,” peers can provide others with health-related information, such as potential interventions or treatments or how to behave or cope in the situation they are facing, and a greater understanding of how others experience the same illness. This is often information that individuals don’t have access to offline [27].

However, users have also identified disadvantages, including the potential for others’ experiences to cause personal distress, restrictive rules and moderation, and unhelpful interactions with others [36]. A survey study exploring adverse events in the use of online peer support found social exclusion and emotional contagion to be other potential risks [37]. Research has also raised the need for guidelines to safeguard vulnerable people, both as users and as moderators of online mental health peer support groups [38]. Furthermore, certain groups, including those with more serious mental illness, may be less likely to engage with the internet, and so may be less inclined to use these groups, missing out on any benefits they provide [39].

## Social Media for Peer Support

Interventions for mental health, such as online peer-to-peer support, have the potential to exploit the increasing use of social media [40,41]. Social media provides users the ability to connect with similar others without fear of stigma or judgement, to create supportive networks and communities to discuss mental health, and to control their own actions, choosing how much to post and interact with others [40,42,43]. Evidence suggests that social media is already being used by individuals with mental illness for support—in a survey of young adults (aged 18-35 years) with a self-identified mental illness, social media was used for sharing personal experiences (68%), connecting with others with a mental illness (66%), learning coping strategies (50%), and learning about mental illness from others (42%) [38].

The most popular social media platform in the United Kingdom is Facebook [9], with 23% of users aged 18 to 24 years and 31% of users aged 25 to 34 years [44]. Recent evidence suggests that Facebook is used by around 90% of people aged 12 to 34 years who identify as having a mental illness [44,45]. Facebook is a free-to-use platform that has similar features to both synchronous and asynchronous platforms, with users able to post content and engage in discussions with others, and the messenger tool allows personal communication with others when group users may not want to share personal stories or information on the main feed. Therefore, Facebook is being increasingly used to host peer-to-peer support groups. These can be private or public, with anyone able to read and post content on public groups, but only those granted access by an individual with administration rights can see content in private groups. Moderators also on occasion set up live chat rooms where users can meet and speak live, on or off camera, with others in the group. Facebook peer support groups for mental health are typically self-organized in an informal way, rather than being organized, run, or moderated by medical professionals.

Currently, there is little research into the use of Facebook groups for mental health peer support. In a content and thematic analysis of public Facebook groups for mental health peer-to-peer support, Prescott and colleagues [46] found that groups are used to share personal experiences, to request advice, to find informational support such as signposting to services, and to seek emotional support. However, this was a passive analysis and did not involve gaining any personal insights, personal information, or confirmation of diagnoses from participants. In a qualitative evaluation of a private Facebook group, Watkins and colleagues [47] found the group to be well-liked by participants, who found it educational and thought-provoking and found that it enabled them to build relationships with others and have conversations they may not have felt comfortable having face-to-face.

## Social Network Site–Based Interventions

As social media has developed as a platform for delivering peer support, some researchers have taken this further to develop a novel form of digital intervention called social network site (SNS)-based interventions. These interventions have an SNS format, combining individually tailored therapy with clinical and peer moderation within a peer-to-peer social support network [48,49]. While initial pilot and small-scale studies have demonstrated positive outcomes following the use of these interventions, further research is required to support their efficacy and to understand how the peer support element can best be utilized to support users.

## Moderation

Many online peer support groups and SNS-based interventions involve moderation, which is important for building a safe and positive community [50]. Moderation within online communities can improve intention to participate [51] and the quality of users’ contributions [52]. Furthermore, moderation has been identified as a key component for the success of online peer-to-peer interventions [53], with the most favorable being those guided by moderators, who are perceived as caring, supportive, and friendly by users.

Previous studies of moderators of various health-related online support groups for both mental and physical health conditions have found that most moderators are individuals living with the condition themselves. Some moderators described how moderating online communities fulfilled their own support needs and reported that it was empowering to be able to help others. Other motivations for setting up groups included addressing a lack of good existing support groups, reducing isolation, and having a place to exchange support and information. Moderators described various roles they took within these groups, such as circulating health information; moderating messages; providing support, encouragement, and advice; making announcements; and performing administrative tasks, such as responding to requests, banning harmful users, and organizing discussion threads [54-56].

In a study specifically examining moderation within mental health communities on Reddit [57], moderators’ motivations for starting Reddit threads included feeling good for helping others, taking the opportunity for leadership, improving the community, and spreading awareness about treatment. Their roles and responsibilities included clearing up spam and troll posts, handling posts on suicide or self-harm, and providing social support. In the same study, Saha and colleagues [57] found that the involvement of medical professionals as moderators or group members could be helpful to answer questions that other group members may have, but it was considered inappropriate to have medical professionals diagnose others via the group.

While moderators have many critical roles within these groups, there may be some inherent risks given they are often vulnerable individuals themselves. Moderation may become overwhelming, taking up much time and energy, and moderators must set boundaries to focus on their own health. Moderators are required to make decisions on censoring potentially harmful content, to which posters may react angrily [36,37]. It is possible that moderators may become stressed with the responsibility of looking after members. Despite these initial studies investigating moderation within online health communities, research has not yet considered the roles and experiences of group moderators within peer-to-peer support groups specifically mediated via Facebook.

## Models and Theories

Several models and theories have been proposed for the mechanisms underlying offline peer support. Gillard and colleagues [58] proposed a change model for peer support worker interventions. Change models provide an understanding of how processes within an intervention are associated with outcomes. The model suggests the primary mechanism is building trusting relationships based on peer workers sharing lived experiences and understanding service users’ experiences. Two parallel mechanisms flow from this: (1) peer workers role-modeling recovery and social functioning and (2) bridging the gap to professional services and the community. The model suggests these mechanisms lead to changes in outcomes such as hope, empowerment, social functioning, self-care, engagement with services, and strength of social networks. Chinman and colleagues [59] suggested these mechanisms show face validity, but no studies have explored their explanatory power. Moreover, no research has investigated whether these mechanisms can be applied to online peer support environments.

It is as yet unclear whether models like these transfer to online peer support, and there is a gap in research in this area [14,59,60]. However, some researchers have set out new models describing what benefits people seek from online networks in particular, and how these benefits may be achieved. Naslund and colleagues [61] proposed a theoretical model for online peer support, incorporating 3 main opportunities provided by such networks. The first, challenging stigma, relates to individuals connecting with similar others and feeling more comfortable expressing themselves due to the anonymity afforded by online peer support networks. The second, increasing consumer activation, suggests online settings enable individuals to learn what to expect from a condition, how to cope with it, and how to approach important health care decisions. The final opportunity suggests that online networks facilitate access to other (online and offline) interventions. No studies have so far empirically tested whether this model is supported by users of Facebook groups for peer-to-peer support.

Aside from these models, various theories from across the discipline of psychology have been suggested to underlie peer support, including social learning theory, social comparison theory, and helper therapy [62]. Social learning theory, in the context of peer support, suggests that peers with a history of or current mental illness act as role models for peers with similar mental illness [63]. Interacting with a peer perceived to be successfully coping with their illness is suggested to be more likely to result in positive behavior change. Barton and Henderson [64] suggested observing another peer demonstrating desired behaviors with visible consequences motivates others to replicate this behavior.

Social comparison theory suggests that people seek out others they perceive as having things in common with, such as a similar mental illness, to establish a sense of normalcy and identity [65]. Interacting with peers who have successfully managed their illness may lead to aspirations of positive behavior change [66]. Upward comparisons, to those perceived to be successfully managing their illness, can provide hope and promote self-improvement. Downward comparisons, to those believed to be worse off, put into perspective how bad things could be and are proposed to be self-enhancing.

Helper therapy proposes that individuals can benefit themselves through helping others [67]. Suggested benefits to the helper include enhanced self-image, development of their abilities, and the status of the helper role influencing the way they are treated [67,68]. Research has highlighted other benefits, including feeling useful to others, reducing internal stigma, feeling looked up to, and having a sense of achievement and competence [69-72]. Helper therapy has been identified as one of the mechanisms underpinning positive behavior changes with peer support-worker interventions [73].

In a study examining the mechanisms of peer support alongside a web-based psychoeducational program, Proudfoot and colleagues [74] found evidence for social comparison theory, which promotes hope, motivation, and faith in treatments, and helper therapy, which helps provide others awareness of how to manage their illness and promotes feelings of competence and a sense of connectedness to the mental health system. However, this study investigated peer support alongside another intervention, and research has yet to explore the mechanisms underpinning behavior and attitude changes in peer support when it is delivered alone in online peer support settings.

When considering how these models and theories may be applied to online peer support, it will also be important to consider the extent to which users engage with and contribute to online groups. A common measure is frequency of posts, which has been suggested to broadly fit the 90-9-1 principle, stating that a majority of content will be posted by a small proportion of users (1%), while the vast majority of users (90%) choose to observe rather than participate actively [75]. However, this is a restricted, unidimensional measure, and reviews of multidisciplinary participatory styles have suggested there are up to 41 different styles of engagement found across various online health communities [76]. Further research is required to understand engagement categories specifically within mental health support groups on Facebook and how this engagement ties into the potential mechanisms of online peer support that have been proposed.

## Research Perspectives

As we emerge into the aftermath of the coronavirus pandemic, it is important now to understand and act on the toll that the pandemic has taken on mental health. There are indications that COVID-19 can have a direct impact on mental well-being through mental and neurological manifestations, with many of those hospitalized with COVID-19 experiencing anxiety or depression [77]. The pandemic also impacted on mental well-being indirectly through grief for those dying as a result of contracting the virus, heightened loneliness, and isolation from social distancing and lockdown measures, as well as anxiety and uncertainty about the future [78]. Financial insecurity will have contributed to poorer well-being for many, with the number of those claiming benefits due to unemployment rising [79], food insecurity quadrupling, and an estimated 1.1 million people at the end of 2020 facing poverty [78]. Young people in particular have seen their mental health significantly worsen over the course of the pandemic [80].

Research shows promise for the use of online SNSs, including Facebook, to provide forums where mental health peer support can take place, with a variety of theories that could plausibly underpin their use. Novel online peer support platforms making use of social network functions may therefore offer a useful way to support people with mental health difficulties. However, it is currently difficult to use or promote online peer-to-peer support as an intervention when we do not have a quantification of the effectiveness of peer support provided in this way or a clear understanding of the benefits its users experience. We argue that in order for existing social network platforms like Facebook to be recommended as peer-to-peer support interventions and for separate peer support interventions to be developed based on SNSs, more research is needed to understand their effectiveness, making use of both quantitative and qualitative methods.

We suggest that the effectiveness of novel social network–based peer support interventions should be evaluated using similar experimental designs as applied in prior research into offline peer support [16,32-34] with a primary focus on symptomology (using both clinical and self-reported outcomes) and psychosocial outcomes, such as self-efficacy in managing disorders, quality of life, hope, knowledge, empowerment, and social isolation. Qualitative evidence could usefully be gathered on existing peer support groups on Facebook to describe processes involved in online peer support, such as how groups are created, how they function, and how groups differ in terms of purpose and target audience.

It is important also to be mindful of the limitations and potential harms of online peer support. The use of peer support may result in adverse events, such as behavioral contagion, corumination, or unpleasant or negative interactions with other users [36,37,81-83]. There is a need to apply principles of responsible research and innovation to future initiatives to develop online peer support, to anticipate potential harms and consequences of these approaches, and to engage with users to surmount these issues wherever possible [84].

Finally, exploring the mechanisms that underlie effective online peer support is crucial to understanding why it is helpful, and how its utility can be maximized. Particularly, in order to develop new interventions that are based on sites like Facebook, that is, SNS-based interventions, it will be important to understand whether theories underpinning offline peer support also apply to online peer-to-peer support in this context. It is also crucial to understand the role of moderation within these communities, and the benefits that moderators have for the workings of online peer support, including safeguarding, to clearly demonstrate their role in future social network–based peer support interventions.

Deriving knowledge from existing online peer support groups (ie, those on Facebook) will be beneficial for the design of new online peer support interventions. Research in this area has the potential to provide a better understanding of why and how these networks help, and how they can best be organized and managed for maximum effect while reducing risk of harms.

## Abbreviations

CMD: common mental disorder
ISG: internet support group
SNS: social network site
